# *Arabidopsis* ZUOTIN RELATED FACTOR1 Proteins Are Required for Proper Embryonic and Post-Embryonic Root Development

**DOI:** 10.3389/fpls.2019.01498

**Published:** 2019-11-22

**Authors:** Donghong Chen, Qiannan Wang, Jing Feng, Ying Ruan, Wen-Hui Shen

**Affiliations:** ^1^Institut de Biologie Moléculaire des Plantes (IBMP), UPR2357 CNRS, Université de Strasbourg, Strasbourg, France; ^2^State Key Laboratory of Subtropical Silviculture, Zhejiang A&F University, Hangzhou, China; ^3^College of Bioscience and Biotechnology, International Associated Laboratory of CNRS-Fudan-HUNAU on Plant Epigenome Research, Hunan Agricultural University, Changsha, China

**Keywords:** ZRF1, root meristem, embryogenesis, cell layer organization, cell identity

## Abstract

The H2A/UBIQUITIN-binding proteins AtZRF1a/b have been reported as key regulators involved in multiple processes of *Arabidopsis* plant growth and development. Yet, the cellular and molecular mechanisms underlying the mutant phenotype remain largely elusive. Here we show that loss-of-function of *AtZRF1a/b* causes defective root elongation and deformed root apical meristem organization in seedlings. The premature termination of the primary root in the *atzrf1a;atzrf1b* double mutant is associated with an advanced onset of endoreduplication and subsequent consumption of reservoir stem cells. Cytological analyses using cell type-specific markers and florescent dyes indicate that *AtZRF1a/b* are involved in maintenance of proper cell layer organization, determinacy of cell identity, and establishment of auxin gradient and maximum at the root tip. During embryogenesis *AtZRF1a/b* act dominantly in regulating the maintenance of ground tissue initial cells and production of lateral root cap. Lastly, quantitative real-time polymerase chain reaction analysis shows mis-expression of some key genes involved in regulating cell patterning, cell proliferation and/or hormone pathways. Our results provide important insight into *AtZRF1a/b* function in cell fate determinacy and in establishment and maintenance of proper stem cell reservoir during embryonic and post-embryonic root development.

## Introduction

The *Arabidopsis* root has a well-organized structure with simple longitudinal organization and few well-defined cell lineages, providing an excellent model system to investigate asymmetric cell division and cell fate determinacy. Root development relies on the root apical meristem (RAM), which not only maintains stem cell self-renewal but also provides different types of daughter cells, which subsequently undergo expansion to form elongation zone and then differentiation to form root hair zone (maturation zone). The balance between cell division and cell differentiation determines RAM size. The RAM consists of the proliferation domain with high dividing-cell content and the transition domain with low dividing-cell content along the longitudinal axis (Ivanov and Dubrovsky, 2013). In *Arabidopsis* (*Arabidopsis thaliana*) root proliferation domain, four types of initial stem cells surrounding approximately four quiescent center (QC) cells (Scheres et al., 1994), together constitute the stem cell niche (SCN). At the distal region, columella cells are generated from anticlinal divisions of the columella stem cell initials, and epidermal cell and lateral root cap are derived from sequentially anticlinal and periclinal divisions of their common epidermal/lateral root cap initials. At the proximal region, cortex and endodermis are derived from the periclinal division of their common ground tissue initials, and stele is formed from the stele stem cell initials. Within the root SCN, QC cells with slowly mitotic activity provide a reservoir for maintenance and replenishment of the surrounding initial stem cells, which exhibit high frequency of cell divisions (Heyman et al., 2013). These ascribed basic RAM cell pattern has been originally established during embryogenesis and maintained during postembryonic primary root growth (Dolan et al., 1993; Scheres et al., 1994).

Transcription factors and phytohormone auxin play critical roles in regulating RAM maintenance and stem cell homeostasis (Drisch and Stahl, 2015). ETHYLENE RESPONSE FACTOR115 (ERF115) as a rate-limiting factor of QC divisions is expressed in dividing QC cells, but it is usually restrained through proteolysis by the APC/C^CCS52A2^ ubiquitin ligase in normal condition (Heyman et al., 2013). WUSCHEL-RELATED HOMEOBOX 5 (WOX5) as one of the most important root stem cell regulatory factor is specifically expressed in QC cells, necessary for the maintenance of undifferentiated state of surrounding stem cells (Sarkar et al., 2007). The CLAVATA3/EMBRYO SURROUNDING REGION (CLE) peptide CLE40 from columella cells is perceived *via* its receptors ARABIDOPSIS CRINKLY4 (ACR4) and CLAVATA1 (CLV1) to modulate the expression level and positioning of *WOX5* (CLE-WOX5 feedback loop), consequently regulating columella stem cell fates (Stahl et al., 2009; Stahl et al., 2013). It is known that auxin signal and distribution are widely involved in root patterning and polarity, SCN maintenance, and distal stem cell identity (Sabatini et al., 1999; Friml et al., 2002; Ding and Friml, 2010). In fact, auxin signal and transcriptional factor usually function in a coordinate way. For instance, WOX5 action is balanced through the activity of indole-3-acetic acid 17 (IAA17) auxin response repressor, together forming WOX5–IAA17 feedback circuit, essential for the maintenance of auxin gradient in RAM and the auxin-mediated columella stem cell differentiation (Tian et al., 2013; Tian et al., 2014).

Polycomb Group (PcG) proteins are conserved in animals and plants, mainly constitute Polycomb Repressive Complex1 (PRC1) and PRC2, acting as key epigenetic regulators during organism growth and developmental processes (reviewed in (Hennig and Derkacheva, 2009; Xiao and Wagner, 2015; Forderer et al., 2016). In Arabidopsis, PcG proteins play essential roles in root development. PRC2 components CLF, SWN, EMF2, VRN2, and FIE are involved in root meristem development and vascular cell proliferation in the maturation zone (Aichinger et al., 2011; de Lucas et al., 2016). CLF also associates with EMF2 to repress founder cell establishment during lateral root initiation associated with down-regulation of root auxin maxima (Gu et al., 2014). Additionally, PRC2 deficiency gives rise to mitotic reactivation and somatic embryogenesis in terminally differentiated root hairs (Ikeuchi et al., 2015). Consistently, the PRC1 subunits AtRING1a/b and AtBMI1a/b/c inhibit the formation of *pkl*-type root-phenotype displaying embryonic traits in primary root mainly through preventing an ectopic expression of embryonic master regulators (Bratzel et al., 2010; Chen et al., 2010). The AtBMI1-interacting factors VAL1/2 also have a similar function (Hoppmann et al., 2011; Yang et al., 2013).

The ZUOTIN-RELATED FACTOR (ZRF) proteins in eukaryotes constitute a novel clade of HSP40 family, which in general serves as co-chaperone of HSP70s to assist protein translation, folding, unfolding, translocation and degradation (Chen et al., 2014). However, the human ZRF1 was found to compete with and replace PRC1 RING1B from chromatin *via* competitively binding H2Aub1 mark, and to favor H2Aub1 removal *via* recruiting the specific deubiquitinase USP21, consequently leading to repressive-to-active chromatin state switch (Richly et al., 2010). In *Arabidopsis*, two ZRF1 homologs, AtZRF1a and AtZRF1b, showed redundant functions. AtZRF1b can bind ubiquitin *in vitro* and pull-down H2Aub1 and H2A from plant protein extracts (Feng et al., 2016), which is in agreement with the human ZRF1 acting as a H2Aub1 reader. The *AtZRF1a/b* genes display broad expression pattern, but with higher levels in the dividing cell-enriched tissues, e.g. meristem, floral bud, and developing embryo. Loss-of-function of *AtZRF1a/b* causes pleiotropic abnormalities including delayed seed germination, plant dwarfism, formation of multiple ectopic meristems, and defects in flower development and gametophyte transmission as well as embryogenesis (Feng et al., 2016; Guzman-Lopez et al., 2016). The *atzrf1a;atzrf1b* mutant displays severely disrupted root developmental phenotype; yet, the underlying mechanism is far from clear.

In this study, we investigated in detail the root developmental defects of the *atzrf1a;b* mutant. We demonstrated that *AtZRF1a/b* play crucial roles in regulating stem cell activity, cell layer organization, cell fate determinacy and cell division orientation in RAM during embryonic and post-embryonic root development.

## Materials and Methods

### Plant Materials and Growth Conditions

The *atzrf1a-1;atzrf1b-1* and *atzrf1a-2;atzrf1b-1* double mutants (Feng et al, 2016) and some marker lines *CYCB1;1:Dbox-GUS* (Colon-Carmona et al., 1999), *WOX5*::*erGFP* (Blilou et al., 2005), *SCR::SCR-YFP* (Heidstra et al., 2004), *CO2*::*H2B*-*YFP* (Heidstra et al., 2004), *DR5rev*::*GFP* (Friml et al., 2003) have been described previously. The Haseloff enhancer trap *GFP* lines J1092 (N9147) and J2341 (N9118) were obtained from the Arabidopsis Biological Resource Center (http://www.arabidopsis.org) or the European Arabidopsis Stock Centre (http://arabidopsis.info). The different reporter lines were respectively introgressed by crossing and homogenized by backcrossing two generations with *atzrf1a-1;atzrf1b-1* double mutant. The genotyping of *atzrf1a-1* or *atzrf1b-1* mutation sites was performed as described (Feng et al, 2016). The existences of reporter genes were detected by directly GUS staining or fluorescent observation. Homozygous *atzrf1a-1;atzrf1b-1* mutant harboring each reporter gene was used for further analysis, and WT harboring the corresponding reporter was set as control. Seeds were surface sterilized (70 and 95% ethanol for 10 min, respectively) and plated on MS medium (MS salts, 0.9% sucrose, pH 5.7, 0.9% bactoagar). After stratification at 4°C for 2 days in the dark, seeds were transferred to a growth chamber at 22°C in a 16/8 h light/dark regime. After 2 weeks, seedlings were transferred to soil at the same condition.

### Phenotypic Analysis

For root length comparison, mutant and control plants were grown side by side on a same plate. Root length was measured from the root tip to the root/hypocotyl border of vertically grown seedlings *via* ImageJ software. The lengths of RAM, proliferation domain and transition domain were measured according to the defined criteria (Napsucialy-Mendivil et al., 2014). In mature embryo, the number of cortex cells was counted in a cell file extending from QC to hypocotyl rootward border; the maximum number of columella cell layers was counted in the columella cell cap including initials. All the above experiments were repeated three times (mean ± SE), each repeat containing at least 15 plants.

### Histology and Microscopy

GUS staining assay was performed as described (Chen et al., 2010). Briefly, whole transgenic marker seedlings were sequentially treated by fixative cold acetone for 30min, and GUS staining buffer (100 mM phosphate buffer, pH 7.4, 2 mM ferricyanide, and 0.5 mM ferrocyanide, and 4 mM X-Gluc) at 37°C in the dark for 4 h. Samples were cleared overnight in 90% lactic acid and were photographed with a differential interference contrast microscope (Leica). For whole-mount visualization, the roots were directly cleared in chloral hydrate solution (chloral hydrate/glycerol/H_2_O, 8/2/1, m/v/v). For starch granule staining, roots were stained with Lugol solution (Chen et al, 2010). For GFP/YFP observation, the roots were counterstained with 20 µg/ml of propidium iodide. For RAM observation, mature embryos and root tips were stained *via* the mPS-PI method (Truernit et al., 2008).

For young embryo observation, embryos at different stages were dissected from developing seeds with tweezers and fine syringe needles, and either were stained with newly-developed cell-wall Dye SCRI renaissance 2200 (SR2200) as described (Musielak et al., 2015) or were cleared by chloral hydrate solution for differential interference contrast observation.

All the above experiments were repeated three times, each repeat containing at least 10 plants.

### Quantitative Real-Time Polymerase Chain Reaction

Total RNA was isolated from 7-day-old roots using the NucleoSpin RNA Plant kit (Macherey-Nagel, Germany). First strand complementary DNA synthesis was carried out using Improm-II reverse transcriptase (Promega). Quantitative real-time polymerase chain reaction (qRT-PCR) was performed on a light cycler 480II (Roche) according to the manufacturer’s instructions. Reaction volumes (10 µl) were comprised of 5 µl of 2× LightCycler 480 SYBR Green I Master (Roche), 2 µl of primer mix, 1 µl of template complementary DNA, and 2 µl of ddH_2_O. Reactions were performed at 95°C for 10 min, followed by 40 cycles of 95°C for 10 s and 60°C for 30 s. The specific PCR product was validated through melting curve analysis. *PP2A* and *EXP* was used as internal reference. Data represent mean values of three technical replicates on three independent biological repeats. The primers for qRT-PCR are listed in **Supplementary Table S1**.

### Flow Cytometry

Nuclei were prepared from roots of 1-week-old plants and analyzed on an Attune^™^ Acoustic Focusing Cytometer (Applied Biosystems, USA). Typically, 10,000 nuclei per sample from at least 100 WT roots and 200 mutant roots were analyzed. Data represent mean values of three technical replicates on three independent biological repeats. Endoreduplication index (EI) which represents the average number of endocycles undergone by a typical nucleus (EI = [0 · n2C + 1 · n4C + 2 · n8C + 3 · n16C]/[n2C + n4C + n8C + n16C]) was calculated as published (Barow and Meister, 2003; Vanstraelen et al., 2009).

## Results

### Loss of AtZRF1a/b Causes Primary Root Growth Arrest

**Figure 1:**
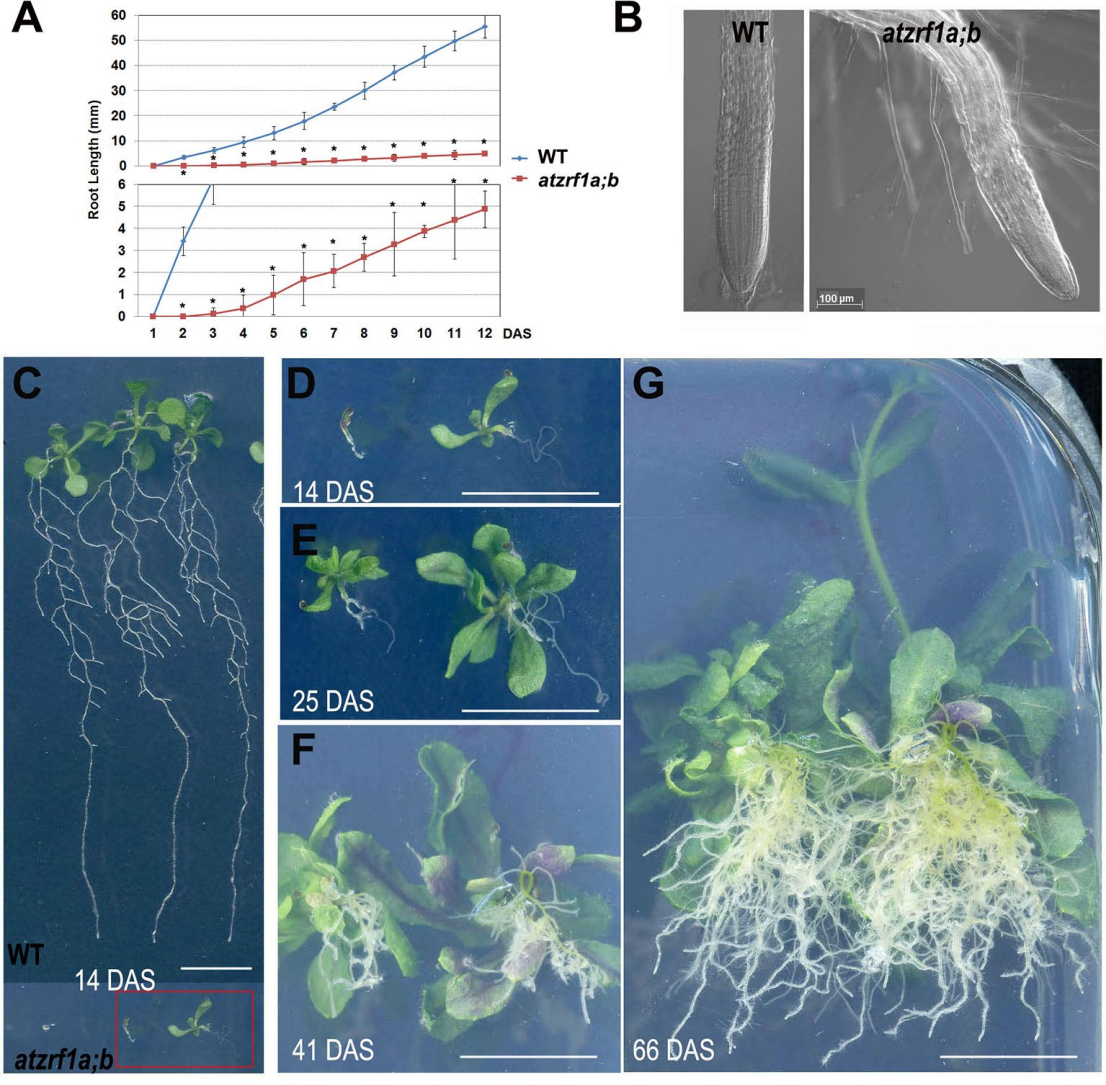
Defective primary root development in the *atzrf1a;b* mutant. **(A)** Comparison of primary root growth between the wild type (WT) and the *atzrf1a;b* mutant plants during 12 days after stratification (DAS). Histogram at the bottom shows a magnification of the Y-axe scale to better view the time course of the *atzrf1a;b* mutant growth. Data represent mean values of three technical replicates on three independent biological repeats. Asterisk indicates student’s *t*-test statistically significant differences at *P* < 0.01. **(B)** Representative images of the WT root tip and the *atzrf1a;b* mutant root tip at 5 DAS. **(C)** Representative WT seedlings showing continuous primary root growth and proliferation of lateral roots at 14 DAS. **(D**–**G)** Representative seedlings of *atzrf1a;b* showing primary root growth arrest and adventitious root development at 14, 25, 41, 66 DAS. Bars = 50 μm in **(B)**, and 1 cm in **(C)** to **(G)**.

The *atzrf1a-1;atzrf1b-1* and *atzrf1a-2;atzrf1b-1* double mutants displayed the same defective root phenotype, whereas WT and *atzrf1* single mutants displayed the normal root growth (**Supplementary Figure S1**). Thus, the *atzrf1a-1;atzrf1b-1* (briefly as *atzrf1a;b*) mutant was chosen for further analysis in this study. The *atzrf1a;b* mutant showed a drastically reduced root growth rate (**Figure 1A**), leading to an extremely short-root phenotype, e.g. only ∼2 mm in length for the mutant roots as compared to ∼20 mm for the WT roots at 7 days after stratification (DAS). The mutant root displayed mature zone characteristically covered by root hairs that arise in close proximity to the root tip (**Figure 1B**), indicating a drastic reduction of the meristem and elongation zone as well as developmentally advanced cell differentiation. The mutant primary roots ceased to grow as early as at 14 DAS (**Figure 1D**), whereas the WT primary roots continuously grew and produced lateral roots (**Figure 1C**). Later on, the mutant plants produced many adventitious roots (**Figures 1E–G**), which sustain plant growth in water and nutrition acquisition.

### The *atzrf1a;b* Mutant Root Exhibits Cell Division Arrest and Precocious Cell Differentiation

**Figure 2:**
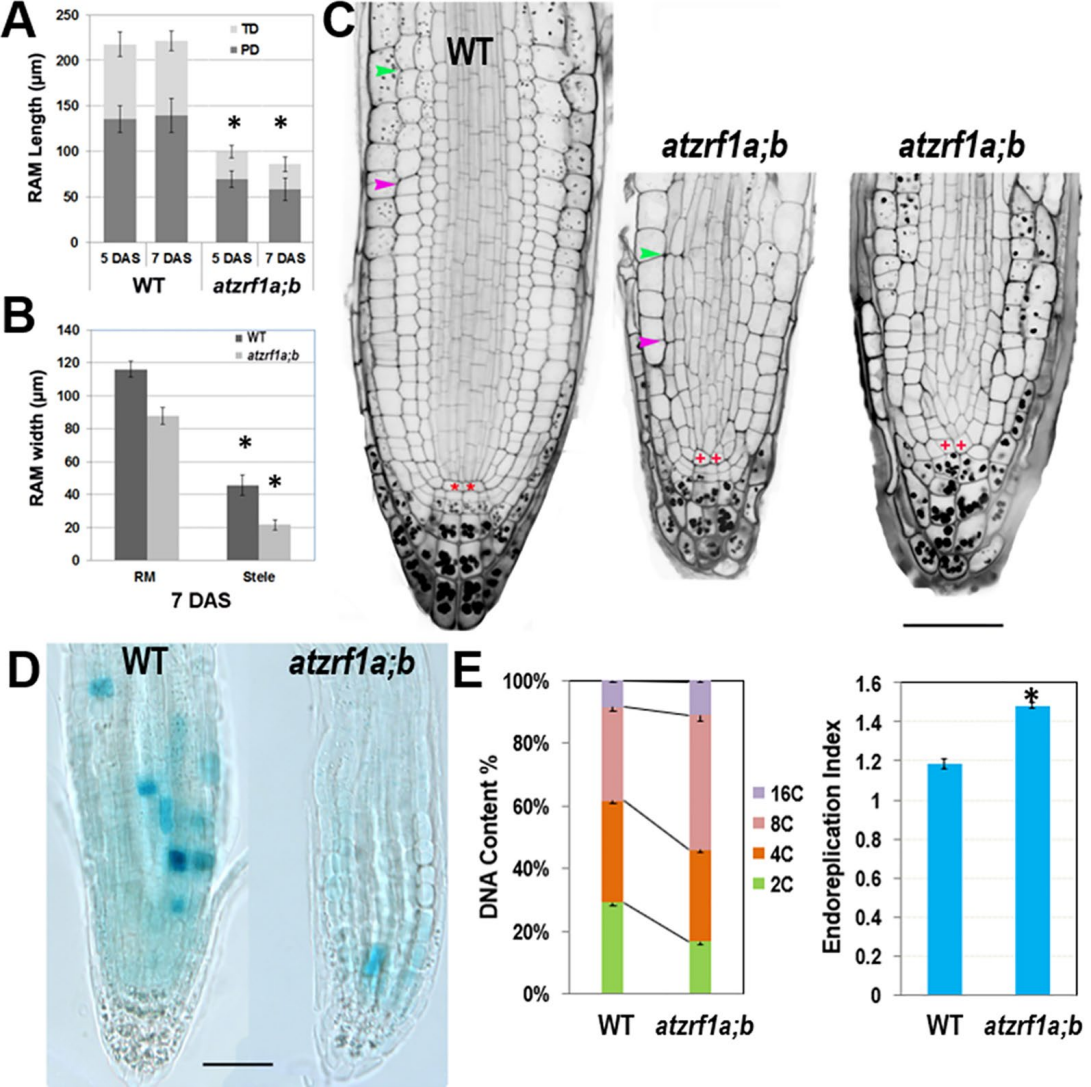
Defective root development is associated with cell division arrest and precocious cell differentiation in the *atzrf1a;b* mutant. **(A, B)** Comparison of meristem size between the wild-type (WT) control and the *atzrf1a;b* mutant at 5 or 7 days after stratification (DAS). The length of RAM including proliferation domain (PD) and transition domain (TD) indicates the vertical distance from QC to PD distal border, and finally to TD distal border. RAM width indicates the diameter at TD distal position. **(C)** 5-day-old RAM in WT and *atzrf1a;b* after mPS-propidium iodide staining. Starch granule is visible in dark and accumulates in the root cap. Pink and green arrowheads indicate the distal borders of PD and TD, respectively. Asterisk indicates position of QC cells. Bar = 50 µm. **(D)** GUS activity of *CYCB1;1::Dbox-GUS* reporter in 5-day-old WT and *atzrf1a;b* mutant. Blue staining indicates for positive GUS activity. Bar = 50 µm. **(E)** Ploidy analysis in 7-day-old WT and *atzrf1a;b* mutant roots. Percentages of 2C, 4C, 8C, and 16C DNA content nuclei are shown. Data show means ± SE from three biological repeats. Asterisk indicates student’s *t*-test statistically significant differences at *P* < 0.01.

Hereinafter we focused on primary roots to investigate *AtZRF1a/b* function. Root growth largely depends on the RAM activity in which cells undergo mitotic cell division, cell expansion and cell differentiation. RAM size is relatively fixed in WT and constantly maintained by the dynamic balance between cell proliferation and cell differentiation. We found that RAM including proliferation domain and transition domain in *atzrf1a;b* is significantly shorter than that in the WT control (**Figures 2A, C** and **Supplementary Figure S2**). Sometimes, the cell arrangement was largely disorganized in *atzrf1a;b* RAM, so that it was hardly distinguished among different cell types (**Figure 2C** and **Supplementary Figure S3**). The root diameters in mutant became evidently narrow mainly due to thinner stele (**Figures 2B, C**). Moreover, the average height to width ratio of the RAM cortical cells in mutant (1.3, n = 30) was higher than that in WT (0.7, n = 30), suggesting cell elongation in advance in mutant RAM. In 3-week-old seedling, the RAM region in mutant was almost completely consumed (**Supplementary Figure S4**). These results indicated that RAM cells in *atzrf1a;b* mutant were undergoing premature differentiation.

In order to investigate the underlying mechanism, we introgressed into the *atzrf1a;b* mutant the *CYCB1;1::Dbox-GUS* reporter which marks the cells at the G2-to-M transition of the cell cycle (Colon-Carmona et al., 1999). Compared to WT, the mutant RAM had reduced GUS staining (**Figure 2D**), which indicates attenuated mitotic activity. On the other hand, we investigated the level of root endopolyploidy, which is associated with cell differentiation. In line with the reduced mitotic activity, EI in *atzrf1a;b* was significantly increased, mainly due to the greatly elevated proportion of 8C and to a less degree of 16C cells (**Figure 2E**), indicating for an early mitosis-to-endocycle transition. Taken together, our data suggest that the decreased mitotic cell division capacity and the advanced onset of endoreduplication lead to reduced RAM size in the *atzrf1a;b* mutant.

### AtZTf1a/b Are Required for Organization and Maintenance of Root Stem Cell Niche

**Figure 3:**
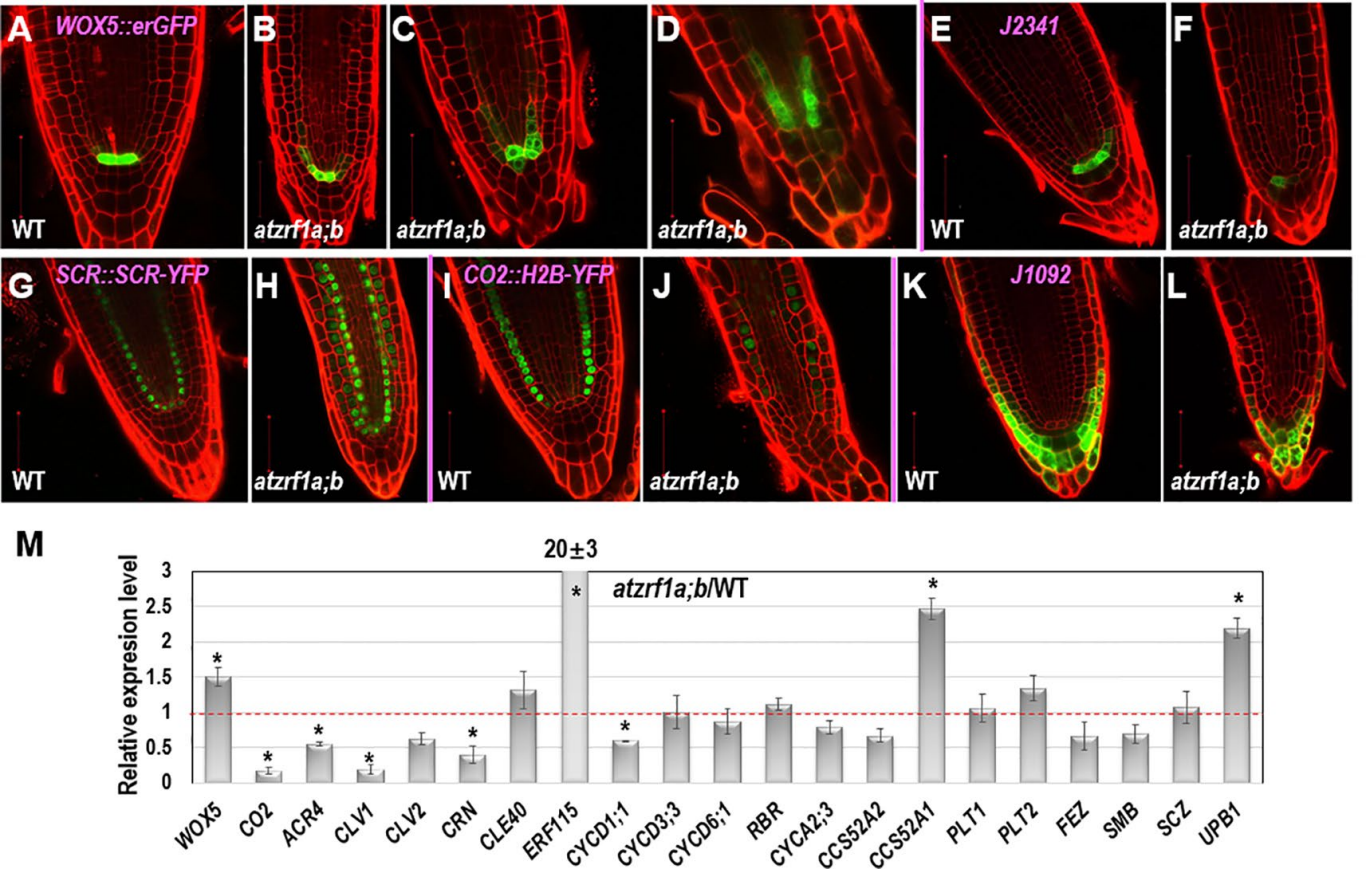
The expression patterns of RAM specific markers in WT and *atzrf1a;b* roots. 5-day-old root was counterstained with propidium iodide and observed with confocal microscopy. **(A**–**D)** *WOX5::erGFP* markers in WT and *atzrf1a;b*. **(E**–**F)** *J2341* markers. **(G**–**H)** *SCR::SCR-YFP* markers. **(I**–**J)** *CO2::H2B-YFP* markers. Inset indicates the relative expression level of *CO2* in 7-day-old root of *atzrf1a;b* compared with that of WT. **(K**–**L)** *J1092* markers. **(M)** Relative expression levels of some RAM-regulating genes in *atzrf1a;b* compared with WT (set as 1) examined by quantitative real-time polymerase chain reaction. Data show means ± SE from three biological repeats. Asterisk indicates student’s *t*-test statistically significant differences at *P* < 0.01. Bars = 50 μm.

To gain insight about cell fate determinacy, root cell-type specific markers were introgressed into the *atzrf1a;b* mutant. Consistent with previous report (Blilou et al., 2005), *WOX5::erGFP* showed specifically expression in QC cells in WT (**Figure 3A**). In contrast, it was found expressed in QC often with aberrant morphology as well as in adjacent cortex/endodermis initial cells in the *atzrf1a;b* mutant (**Figures 3B–D**). The abnormal pattern observed in the mutant might provide inappropriate position cues to surrounding stem cells, as reflected by irregular SCN formation in the mutant (**Figure 2C**). The *J2341* enhancer trap marker carrying ER-tethered GFP (Kim et al., 2005) was found expressed in approximately four columella initials in WT (**Figure 3E**), but was found only expressed in one cell below the QC in the *atzrf1a;b* mutant (**Figure 3F**), indicating weakened columella initial stem cell activity in the mutant. The *SCR::SCR-YFP* endodermis marker (Heidstra et al., 2004) was found expressed in endodermis, cortex/endodermis initials and QC in both WT (**Figure 3G**) and the *atzrf1a;b* mutant (**Figure 3H**). Remarkably, in the mutant, additionally *SCR::SCR-YFP* showed weak but significant expression in cells normally corresponding to the cortex cells (**Figure 3H**). To further verify this mutant defect, we examined expression pattern of *CO2::H2B-YFP*, a marker specific for cortex cells (Heidstra et al., 2004). As expected, *CO2::H2B-YFP* was found expressed specifically in cortex layer cells in WT (**Figure 3I**). In the *atzrf1a;b* mutant, however, only a few cells from the cortex layer showed roughly normal level of *CO2::H2B-YFP* expression whereas the other cells showed low or absence of *CO2::H2B-YFP* expression (**Figure 3J**). These observations using both *SCR::SCR-YFP* and *CO2::H2B-YFP* indicate that the *atzrf1a;b* mutant is impeded in establishment and maintenance of the cortex cell fate during root development. The *J1092* enhancer trap line (Blilou et al., 2002) displayed strong GFP signal in the lateral root cap initial cells and to a lesser extent in the columella root cap initial cells in WT (**Figure 3K**), but showed rather uniform expression level throughout the root cap initial cells in the *atzrf1a;b* mutant (**Figure 3L**), implying a weakened distinction between the two types of root cap initial cells in the mutant. Taken together, our analyses using cell fate markers indicate that *AtZRF1a/b* are required for whole SCN architecture, including QC localization, columella stem cell maintenance, separation between cortex and endodermis identities as well as stable maintenance of cortex cell fate, and distinction between the columella and lateral root cap initials.

Next, we performed qRT-PCR to compare between the *atzrf1a;b* mutant and the WT control for relative expression levels of some of the above described genes as well as others genes known in previous publications as important regulators of root development. As shown in **Figure 3M**, the expression of *WOX5* was upregulated whereas that of *CO2* was drastically downregulated in the *atzrf1a;b* mutant roots. This is in agreement with the *WOX5::erGFP* and *CO2::H2B-YFP* expression pattern described above. Consistent with the *CLE*-*WOX5* feedback repressive pathway, the expression levels of several CLE-reception component genes, i.e. *ACR4*, *CLV1*, *CLV2*, and *CORYNE* (*CRN*) (Miwa et al., 2008; Stahl et al., 2009; Meng and Feldman, 2010; Stahl et al., 2013), were downregulated albeit *CLE40* itself was upregulated in the *atzrf1a;b* mutant roots (**Figure 3M**). In addition, expression of *ERF115*, which is associated with dividing QC cells (Heyman et al., 2013), was found drastically upregulated in *atzrf1a;b*, further indicating defects of QC regulation in the mutant. We then examined expression of several cell cycle regulatory genes known as being involved in root development, including the G1-phase D-type cyclins (*CYCD1;1*, *CYCD3;3* and *CYCD6;1*), the G1-S transition inhibitor *RETINOBLASTOMA*-*RELATED* (*RBR*), the S-phase A-type cyclin *CYCA2;3*, and the endocycle switch regulators *CCS52A1*/*FZR2* and *CCS52A2*/*FZR1* (Vanstraelen et al., 2009; Sozzani et al., 2010; Cruz-Ramirez et al., 2012; Forzani et al., 2014). It was found that *CYCD1;1*, *CCS52A2*/*FZR1* and to a less degree *CYCA2;3* were downregulated whereas *CCS52A1*/*FZR2* was upregulated and the other ones remained unchanged in the *atzrf1a;b* mutant roots (**Figure 3M**). The downregulation of *CYCD1;1* correlates with the high level of *WOX5* in *atzrf1a;b*, which is in agreement with *CYCD1;1* being repressed by WOX5 (Forzani et al., 2014). Lastly, we checked expression of several root-patterning transcription factor genes, including *PLETHORA1* (*PLT1*) and *PLT2* involved in auxin-dependent axial patterning (Aida et al., 2004), *FEZ* and *SOMBRERO* (*SMB*) that antagonistically regulate asymmetric cell division of epidermal and lateral cap initials as well as columella stem cells (Willemsen et al., 2008; Bennett et al., 2014), *SCHIZORIZA* (*SCZ*) that is required for the specification of cortex identity and the separation of cell fates in surrounding RAM layers (Pernas et al., 2010; ten Hove et al., 2010), and *UPBEAT1* (*UPB1*) that functions in the maintenance of cell proliferation-differentiation balance by controlling ROS production (Tsukagoshi et al., 2010). It was found that *UPB1* and to a less degree *PLT2* were upregulated whereas *FEZ* and *SMB* were downregulated in *atzrf1a;b* (**Figure 3M** and **Supplementary Table S2**), implying defects in the regulation of cell fate determinacy and homeostasis between cell proliferation and cell differentiation in the mutant. Taken together, our data indicate that loss of *AtZRF1a/b* perturbs expression of multiple sets of genes involved in diverse pathways in the regulation of postembryonic root development.

### AtZRf1a/b Are Required for Proper Auxin Regulation of Root Development

**Figure 4:**
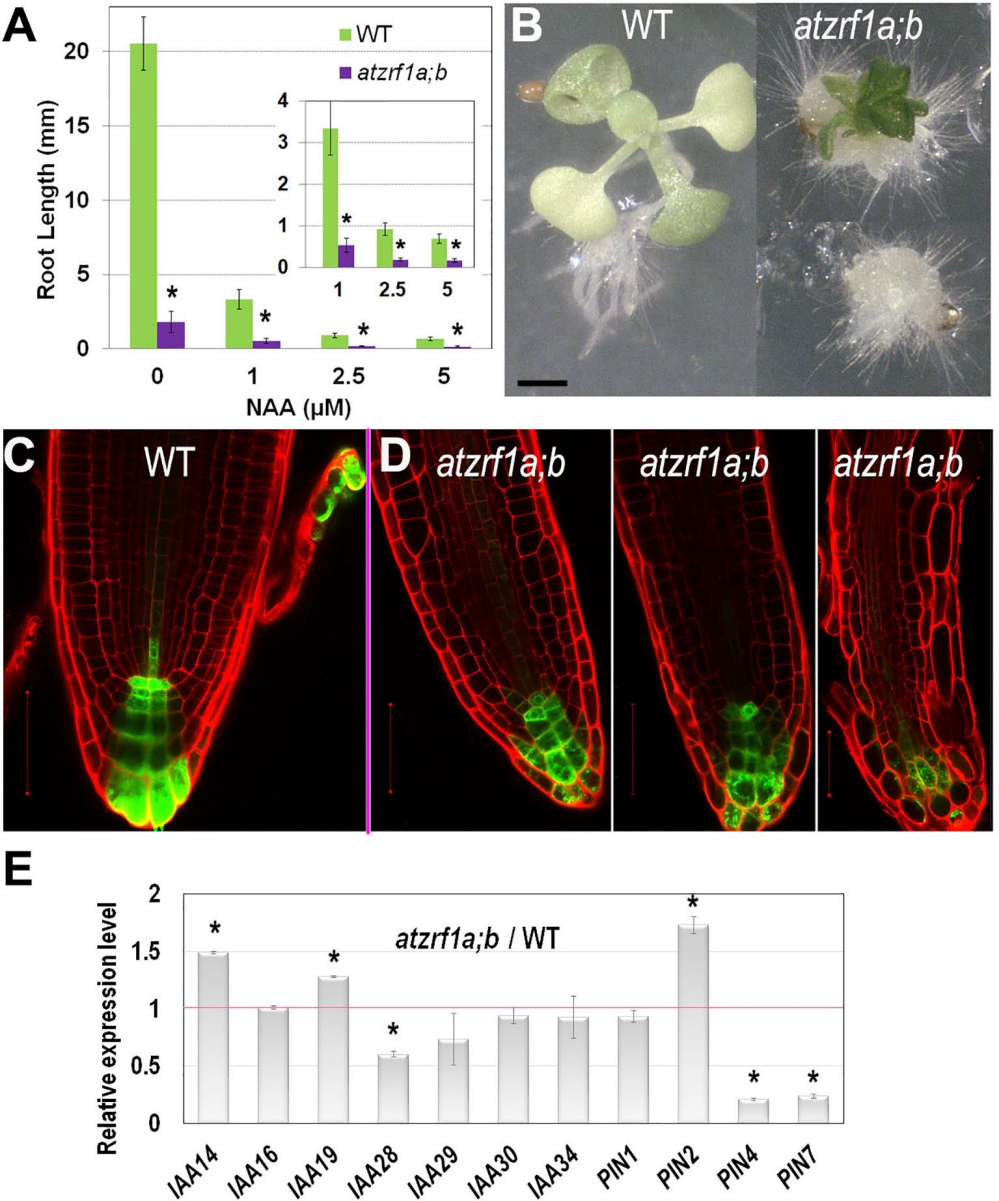
Effect of auxin treatment on *atzrf1a;b* double mutant. **(A)** Effects of exogenous NAA on root growth of WT and *atzrf1a;b* seedlings. Seeds were sown on MS medium containing the indicated concentration of NAA for 1 week. **(B)** WT and *atzrf1a;b* plants grown on the plate containing 5μM NAA in 3 weeks. **(C**–**D)** *DR5rev::GFP* markers in 5-day-old seedlings of WT **(C)** and *atzrf1a;b* **(D)**. Propidium iodide was used to stain the cell wall. **(E)** Relative expression levels of auxin-responsive genes in 7-day-old *atzrf1a;b* compared with WT. Data show means ± SE from three biological repeats. Asterisk indicates student’s *t*-test statistically significant differences at *P* < 0.01. Bars = 1 mm in **(B)**, 50 μm in **(C)** and **(D)**.

Generation and maintenance of auxin gradients and regional maxima in root tip is crucial for normal root development (reviewed in Overvoorde et al., 2010). To survey whether the *atzrf1a;b* mutant abnormal root development is related to any impaired auxin regulation, we first tested plant growth sensitivity to auxin treatment. We found that the root growth of both WT and mutant was seriously inhibited by exogenous 1-naphthalene acetic acid (NAA) increasingly with higher concentrations (**Figure 4A**). The mutant root growth became most completely blocked at the presence of 2.5 µM NAA. When grown at 5 µM NAA for 3 weeks, all the mutant roots and about 10% (N = 50) of whole seedlings developed into callus-like structures with root hairs appeared on the surface whereas WT seedlings never showed callus formation (**Figure 4B**). These data indicate that the *atzrf1a;b* mutant is more sensitive to auxin treatment than WT.

To investigate whether auxin distribution is disturbed in the *atzrf1a;b* mutant RAM, we introgressed into the mutant the auxin-response reporter *DR5rev::GFP* (Friml et al., 2003). In WT, *DR5rev::GFP* was expressed in QC, columella stem cells and columella, displaying auxin gradient maxima in QC and distal columella cells (**Figure 4C**). In the *atzrf1a;b* mutant, the number of cells expressing *DR5rev::GFP* was reduced at varied degrees in individual roots and the auxin gradient maxima at both QC and distal columella cells were lost or weakened (**Figure 4D**). Actually in the cotyledon, a main vein and several secondary veins unite into almost four areoles in WT, but retain unclosed in *atzrf1a;b* (**Supplementary Figure S5**), in consistent with the damaged auxin distribution in mutant. Furthermore, we carried out qRT-PCR to analyze the expression levels of auxin-responsive genes (*IAA14*, *IAA16*, *IAA19*, *IAA28-IAA30*, *IAA34*) and polar auxin transporter genes (*PIN1*, *PIN2*, *PIN4*, *PIN7*), which play important roles in root development (Blilou et al., 2005; Overvoorde et al., 2010). It was found that expression of *IAA14*, *IAA19*, and *PIN2* was increased whereas that of *IAA28*, *IAA29*, *PIN4*, and *PIN7* was decreased in the *atzrf1a;b* mutant roots (**Figure 4E** and **Supplementary Table S3**). Collectively, our data indicate that defects in auxin signaling, transport and/or cell type-specific distribution contribute partly to the *atzrf1a;b* mutant root phenotype.

### AtZRf1a/b Regulate Cell Division and Cell Patterning in Embryonic Root

**Figure 5:**
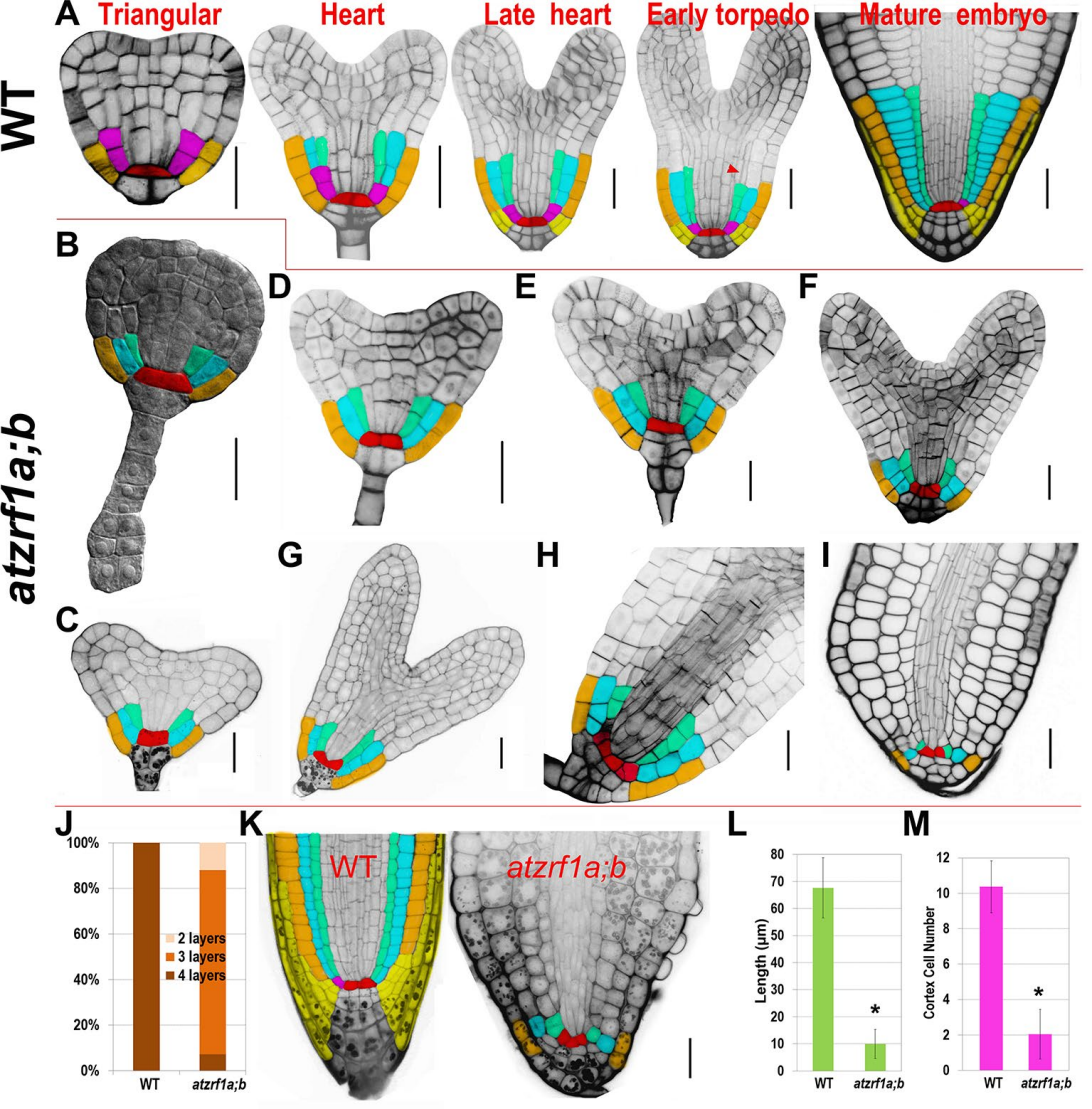
Defective embryogenesis in *atzrf1a;b* mutant. **(A)** Representative embryogenetic stages in WT according to previously reported (Scheres et al., 1994). **(B** *–* **I)** Embryogenesis in *atzrf1a;b*. **(B)** in triangular stage, **(C**–**E)** in different heart-stages, **(F**–**H)** in torpedo stages, and **(I)** in mature embryo stage. **(J)** Columella cell layers of mature embryos in WT and *atzrf1a;b*. **(K)** Three-day-old RAM in WT and *atzrf1a;b* mutant observed *via* mPS-propidium iodide method. **(L)** Length of mature embryonic roots in WT and *atzrf1a;b*. **(M)** Cell numbers in a single file of cortex of mature embryos in WT and *atzrf1a;b*. **(A)** and **(D**–**I)** were stained with SR2200 for confocal observation (Musielak et al., 2015); **(B)** was cleared by chloral hydrate solution for differential interference contrast observation; **(K)** was obtained by the mPS-propidium iodide method (Truernit et al., 2008). Red, QC; pink, ground tissue initial; cyan, cortex; green, endodermis; brown, epidermis; yellow, lateral root cap and its initials. Arrowhead, the second formative cell division giving rise to the second cortex file. Data show means ± SE from three biological repeats. Asterisk indicates student’s *t*-test statistically significant differences at *P* < 0.01. Bars = 20 µm.

To trace the embryonic origin of defective RAM formation in the *atzrf1a;b* mutant, embryos at different developmental stages were analyzed using the cell-wall fluorescent dye SR2200, which had been previously demonstrated to be powerful for investigation of early stages of embryogenesis (Musielak et al., 2015). In globular stage, the extra-proembryo-derived hypophysis located at the uppermost suspensor cell underwent an asymmetric division to produce an upper lens-shaped QC cell and a lower columella initial in both WT and *atzrf1a;b* without showing significant difference (**Supplementary Figures S6A–D**). In triangular stage, ground tissue initial in WT performed a typical anticlinal division to maintain self-renewal and at the same time create a daughter cell which subsequently underwent a periclinal asymmetric division (first formative division) to generate a cortical initial and an endodermis initial (**Figure 5A**). However, the potential ground tissue initial in *atzrf1a;b* seemingly bypassed the former anticlinal division and directly underwent the first periclinal division to give rise to the presumptive cortex and endodermis (**Figure 5B**) and absence of obvious ground tissue initial after this division (**Figures 5C–I** and **Supplementary Figures S6E–G**). In late heart stage, an additional cortical layer arises from the secondary formative divisions of endodermal cells in WT (**Figure 5A**), demarcating the boundary between root and hypocotyl (Bougourd et al., 2000). Similarly, another periclinal division in mutant also happened in the inner ground tissue cell (**Figure 5F**). In the early torpedo stage, the lowest of the protoderm cells in WT formally served as epidermal/lateral root cap initials characterized by the emergence of lateral root cap due to the periclinal division (**Figure 5A**. However, the corresponding protoderm cells in mutant lacking the hallmarking periclinal division failed to achieve the cell fate transition and to generate lateral root cap (**Figures 5F–I**). These indicate *AtZRF1a/b* are necessary for the formative cell division giving rise to epidermal/lateral root cap initials and lateral root cap. QC cells in WT are mitotically quiescent and were transversely aligned in the center of embryonic RAM throughout the whole embryogenesis (**Figure 5A**). In comparison, QC cells in mutant were also easily recognized prior to the heart stage even their morphology gradually growing abnormal from trapezoid to triangle, and then inclined to become atypical in later stages due to their active and irregular divisions giving rise to ill-organized patterning (**Figures 5G–I**). Consistently, the columella cell and columella initial exhibited anatomic defects to a different extent in *atzrf1a;b* mutant (**Figures 5H, I** and **Supplementary Figures S6E–G**). In mutant mature embryo, columella initial and columella cell in *atzrf1a;b* mutant displayed distorted and reduced number of cell layers, with 82% of mutants showing three layers instead of four in WT (**Figures 5A, I, J**). Consistently, 3-day-old *atzrf1a;b* seedlings still have three layers of columella cells, less than WT with five layers (**Figure 5K**). Additionally, the embryonic radicle length in *atzrf1a;b* mutant (∼10 µm) was only about one seventh of that in WT during mature embryo stage, giving rise to a round end phenotype (**Figure 5I, L**). Correspondingly, the mutant radicle had only ∼2 cells in a longitudinal cortex file, much fewer than WT with ∼10 (**Figure 5M**), indicating that AtZRF1a/b promote cell division during embryonic root morphogenesis. Suspensor is comprised of a single file of about seven cells, bridges the embryo proper to surrounding endosperm tissues, and transports nutrients and growth regulators to the embryo (Kawashima and Goldberg, 2010). As embryo grows up, suspensor gradually degenerates till to disappear. But in mutant, suspensor cells were usually arranged into two files at the basal part (**Figure 5B** and **Supplementary Figure S6H**), and sometimes proliferated into a cell mass (**Supplementary Figures S6I, J**). Even in mature embryos, 32% (n = 50) of suspensors were still visible (**Supplementary Figure S6K**). In summary, *AtZRF1a/b* participate in the proper radicle cell patterning, maintenance of QC and surrounding stem cell identity, promotion of cell division, and normal degradation of suspensor cell during embryonic radicle formation.

### AtZRf1a/b Are Required for Embryonic Root Cell Fate Establishment

**Figure 6:**
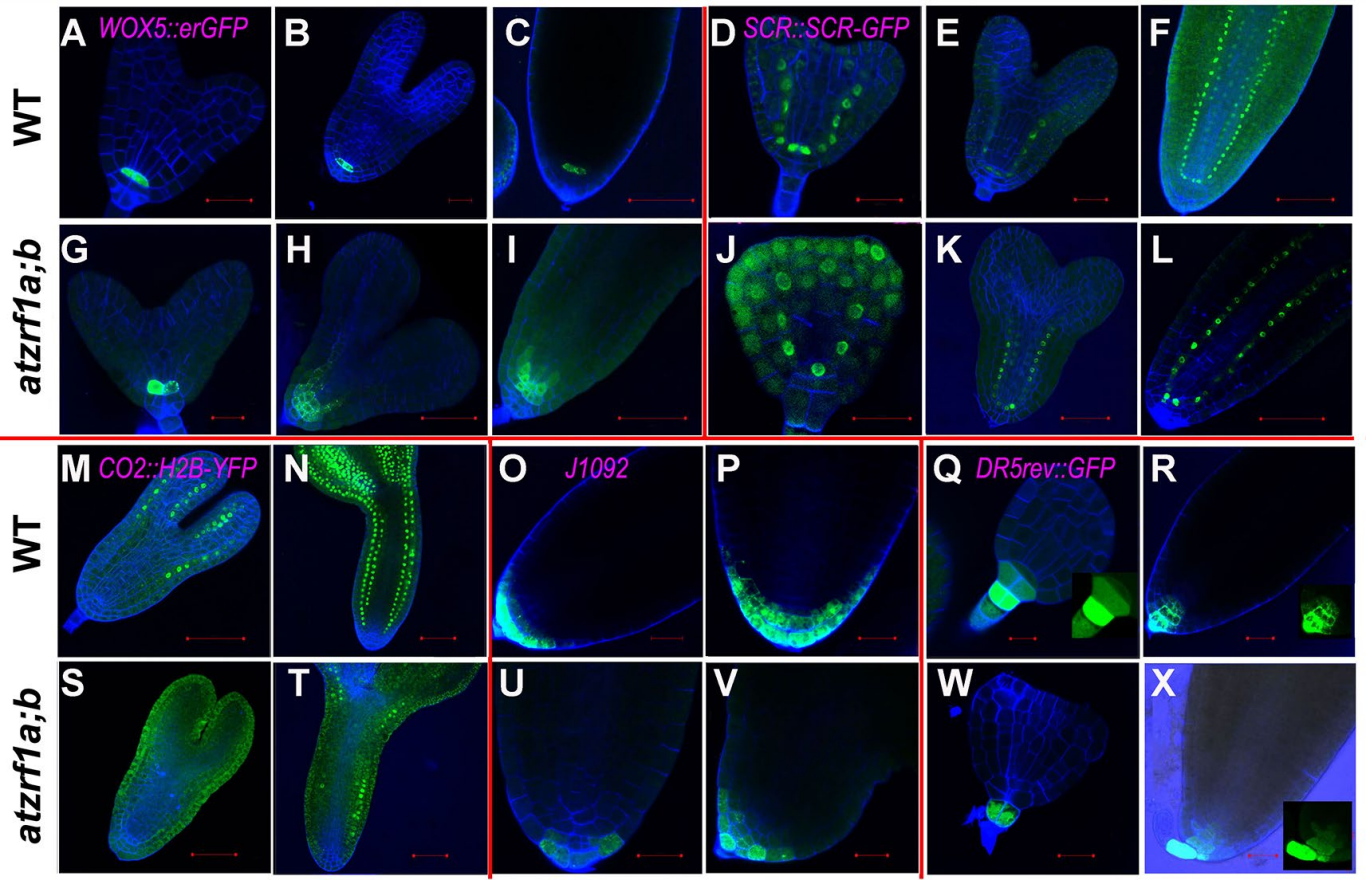
The expression of RAM-specific markers in *atzrf1a;b* embryos. Different stages of embryos were hand-dissected, counterstained with SR2200 and observed under confocal. **(A**–**C)** and **(G**–**I)** *WOX5::erGFP* markers in WT and *atzrf1a;b*. **(D**–**F)** and **(J**–**L)** *SCR::SCR-YFP* markers. **(M**,** N)** and **(S**, **T)** *CO2::H2B-YFP* markers. **(O**, **P)** and **(U**, **V)** *J1092* markers. **(Q**, **R)** and **(W**, **X)** *DR5rev::GFP* markers. Bars = 20 µm, except 50 µm in **(C)**, **(F)**, **(H)**, **(I)**, **(L)**, **(M)**, and **(S)**.

To gain insight into the embryonic root cell identity in *atzrf1a;b*, some aforementioned marker lines were investigated during embryogenesis. QC-specific marker *WOX5::erGFP* also displayed significantly diffused expression in adjacent initials from torpedo stage onward in mutant embryo (**Figures 6A–C, G–I**), consistent with the *WOX5* performance in the postembryonic root. *SCR::SCR-YFP* marked QC, ground tissue initial, and endodermis in embryo as it is in seedling stage in WT. In mutant, *SCR* signal firstly appeared in the inner ground tissue layer from the first unusual periclinal division of ground tissue initial in triangular-stage mutant embryo, confirming the ground tissue inner layer adapted the endodermis cell fate. Subsequently, signal was also observed in the fourth layer from the second periclinal division in mutant embryo, corresponding to the same endodermis layer in WT hypocotyl (**Figures 6D–F, J–L**). *CO2::H2B-YFP* marker was firstly observed in the cortex cells in the upper part of torpedo-stage WT embryo but excluding embryonic root region, and subsequently in all the cortex cells in later stages (**Figures 6M, N**). However, in *atzrf1a;b* mutant, YFP signal was not detected in torpedo-stage, but later was sporadically found in some cortex cells excluding embryonic root region (**Figures 6S, T**), indicating *AtZRF1*a/b are required for determination of cortex cell identity in embryonic root. *J1092* marker specified root cap in the embryos of WT and *atzrf1a;b* mutant, though mutant had no lateral root cap or significantly reduced lateral root cap region in later stage (**Figures 6O, P, U, V**). *DR5rev::GFP* marker had the strongest expression in the nearest suspensor cell attaching to embryo proper in WT and mutant, and subsequently auxin distribution gradients were established from columella cells to QC in WT, but almost absent in mutant (**Figures 6Q, R, W, X**). These data suggested *AtZRF1a/b* are necessary for cell identity maintenance of QC and different initials and the formation of auxin gradients during embryogenesis.

## Discussion

In this study, we provide important insights about the roles of the H2A/UBIQUITIN-binding chromatin regulator genes *AtZRF1a/b* in embryonic and post-embryonic root development. In the loss-of-function *atzrf1a;b* mutant, the primary root growth ceased early during seedling growth because RAM became shortened and exhausted due to spoiled balance between cell proliferation and differentiation. The *atzrf1a;b* RAM displayed low mitotic activities, which was consistent with the very slow root growth. Elevated polyploid levels were detected, indicating an advanced onset of endoreduplication in the mutant roots. In a previous study, endoreduplication levels were also found increased in true leaves of the *atzrf1a;b* mutant plants (Feng et al., 2016). It thus appears that *AtZRF1a/b* repress the mitosis-to-endocycle transition in a general rather than an organ-specific manner.

Proper RAM structure organization is crucial in maintaining continuous post-embryonic root development. Our study showed that *AtZRF1a/b* are crucial in establishment and maintenance of cell fate of various cell types within RAM. The hardly recognizable QC cells together with expanding zone of *WOX5::erGFP* expression outside QC position indicated that the QC cell fate was drastically impacted in the *atzrf1a;b* mutant. In addition, the cell identities of surrounding initials and their corresponding descendants were also altered to different extents in the mutant. Columella root cap was frequently found disorganized, correspondingly, columella initial marker *J2341* displayed reduced activity in the mutant. lateral root cap was clearly separated from columella root cap in WT, but seemingly became undistinguished from columella root cap or absent in the *atzrf1a;b* mutant. Ground tissues including cortex and endodermis partially lost their cell identities, which was reflected by diffused *SCR::SCR-YFP* expression and reduced *CO2::H2B-YFP* signal in the mutant.

RAM defects in *atzrf1a;b* could be traced back to early embryogenesis. The first major defect in *atzrf1a;b* happened in triangular-stage embryo where potential ground tissue initial skipped the first anticlinal division (proliferative division) which was substituted by a periclinal division. The inner ground tissue layer had the endodermis cell identity confirmed by *SCR::SCR-YFP* marker, which was different from that in seedling, whereas the outer ground tissue layer seemed to loss partial cortex cell identity reflected by *CO2::H2B-YFP* marker. The second major defect occurred in late heart-stage embryo, in which the potential epidermal/lateral root cap initial failed to perform the periclinal division (formative division), leading to no lateral root cap formation in late stages. Accordingly, *J1092* marking root cap has reduced expression domains in mutant. So, the *atzrf1a;b* root cap mainly results from columella cell cap but not lateral root cap. In fact, the both major defects abovementioned in mutant were characterized by transformation of cell division orientation (anticlinal-to-periclinal or vice versa). So, *AtZRF1a/b* might be involved in regulating the conversion between proliferative division and formative division rather than specific proliferative or formative division. Additionally, embryonic QC was conspicuous at the beginning, and subsequently conducted a few more divisions even in oblique direction to generate some offspring with similar size and irregular organization, leading to hardly distinguishing from surrounding stem cells in most cases. It seems that *AtZRF1a/b* are required for repression of cell divisions and maintenance of precise division orientation within QC. *WOX5::erGFP* also displayed expanded expression during the late embryogenesis, similar to its performance in seedling root.

The phytohormone auxin play key roles in root development. In WT, local auxin maximum as the prerequisite for QC establishment determines the position of the QC, and the auxin gradient is crucial for maintaining columella initial identity (Sabatini et al., 1999; Tian et al., 2014). In the *atzrf1a;b* mutant, the auxin maximum and/or gradient were perturbed in postembryonic and embryonic roots, where the cell patterning of QC and columella cell was mostly impaired. Accordingly, several *IAA* genes as auxin signal pathway repressors (Fukaki et al., 2002; De Rybel et al., 2010) and *PIN* genes as auxin transporters (Blilou et al., 2005) were misregulated in the mutant roots. Exogenous auxin treatment also showed that *atzrf1a;b* was more sensitive, with enhanced facilities of ectopic callus formation. Future genetic interaction studies may precisely investigate the role and regulatory pathways of auxin in the *AtZRF1*-regulated root development.

In addition to *IAA* and *PIN* genes, several other genes were found deregulated, which likely contributes to the *atzrf1a;b* mutant root developmental defects. Firstly, *AtZRF1a/b* may regulate the balance between cell division and differentiation in RAM partially through CCS52A-activating APC/C ubiquitin ligase. *CCS52A* has two isoforms with antagonistic functions; *CCS52A1* expressed in the root elongation zone promotes endocycle onset and mitotic exit through destruction of A2-type cyclin CYCA2;3 (Boudolf et al., 2009), whereas *CCS52A2* expressed in the RAM distal region controls QC identity and stem cell maintenance (Vanstraelen et al., 2009) through proteolysis of QC division marker ERF115 (Heyman et al., 2013). In *atzrf1a;b*, upregulation of *CCS52A1* coupled with downregulation of *CYCA2;3* was associated with downregulation of *CCS52A2* coupled with upregulation of *ERF115*, which was in line with *ccs52a2* mutant root phenotype displaying the consumed and disorganized RAM (Vanstraelen et al., 2009). Secondly, upregulation of *UPB1* in *atzrf1a;b* was consistent with reduced root length and RAM size in *UPB1* overexpression line (Tsukagoshi et al., 2010). Lastly, CLE peptide ligands in differentiated columella cells regulate *WOX5* expression and columella initial fate through the receptor-like kinases *ACR4*, *CLV1*, *CRN*, and *CLV2* (Miwa et al., 2008; Stahl et al., 2009; Meng and Feldman, 2010; Stahl et al., 2013). *AtZRF1a/b* also regulated RAM organization dependent on *CLE*-*WOX5* pathway inferred from uniformly downregulated expression of *CLE* receptors in mutant. Correspondingly, *ACR4* has an important role in formative cell division and columella cell organization in the root apex (De Smet et al., 2008).

In multicellular organisms, stem cells can maintain self-renewal and produce the new daughter cells with distinct fates by asymmetric cell divisions or formative divisions, which are coordinated by extrinsic and intrinsic cues (Kajala et al., 2014). asymmetric cell division can be considered as the evolutionary engine, leading to cell differentiation necessary for the innovation of novel organ and the emergence of higher life form. Our study demonstrates that the *Arabidopsis AtZRF1a/b* are required for formative division giving rise to lateral root cap during embryogenesis. Likely, *ZRF1* orthologs have an evolutionarily conserved function in asymmetric cell division. For instance, in the classic animal model *Caenorhabditis elegans*, *DNJ11* is involved in the asymmetric division of the neuroblast *via* regulating the orientation of the mitotic spindle (Hatzold and Conradt, 2008). In green algae *Volvox carteri*, *Gonidialess A* (*GlsA*) is necessary for separation of germ and somatic cell fate during gonidium formation (Miller and Kirk, 1999).

*AtZRF1a* and *AtZRF1b* have similar and broad expression in almost all the young plant organs including root tips, shoot tips, developing leaves, inflorescences, floral buds, and embryos; their expression intensity is positively correlated with dividing activities of the organs (Guzman-Lopez et al., 2016). Consistently, loss of *AtZRF1* function results in morphological defects in almost all the developmental phases related to dividing cells and meristematic tissues (Feng et al., 2016; Guzman-Lopez et al., 2016). Recently *AtZRF1a/b* have been reported to perform both PRC1-related and independent functions in regulating plant growth and development (Feng et al., 2016). Consistently, PRC1 RING-finger proteins functioning as H2Aub1 writers and AtZRF1 as H2Aub1 reader share a set of target genes and partial regulatory pathways (Feng et al., 2016). In addition, PRC1 RING-finger proteins display the similar expression pattern and tendency with ubiquitously organic distribution but high levels in dividing cells (Qin et al., 2008; Chen et al., 2010; Chen et al., 2016). Furthermore, *AtRING1a/b* and *AtBMI1a/b* are also widely involved in regulating multiple developmental processes. On the other hand, according to the working model of human *ZRF1* (Richly et al., 2010), *AtZRF1a/b* might also act as a chromatin state switch to remove PRC1 function in the specific developmental context. Future studies are necessary to investigate these different aspects of interplay between *AtZRF1a/b* and PRC1 complexes in the regulation of gene transcription in the root and other plant organ development.

## Conclusion

Our study suggests *AtZRF1a* and *AtZRF1b* participate in controlling root development and morphogenesis through regulating cell proliferation and differentiation, cell identity maintenance, cell patterning, and auxin gradient establishment at the root tip during embryonic and post-embryonic stages.

## Data Availability Statement

The datasets generated for this study are available on request to the corresponding author.

## Author Contributions

DC designed the study, performed most of the experiments, analyzed the data, and wrote the draft. QW and JF performed the introduction of gene marks. W-HS conceived the study and supervised the experiments. W-HS, QW, and YR revised the article. All authors have read and approved the final manuscript.

## Funding

This work was supported by the French Agence Nationale de la Recherche (ANR-08-BLAN-0200-CSD7, ANR-12-BSV2-0013-02), the National Basic Research Program of China (973 Program, grants no. 2012CB910500), the French Centre National de la Recherche Scientifique (CNRS, LIA PER), and National Natural Science Foundation of China (31870310).

## Conflict of Interest

The authors declare that the research was conducted in the absence of any commercial or financial relationships that could be construed as a potential conflict of interest.

## Supplementary Material

Figure S1 The representative root phenotype in 5-day-old atzrf1 single and double mutants. The atzrf1a-1;b-1 and atzrf1a-2;atzrf1b-1 double mutants displayed the same extremely short-root root phenotype, whereas WT and atzrf1 single mutants displayed the normal root growth. Bar = 1 cm.

Figure S2 7-day-old root phenotype in WT and atzrf1a;b mutant via DIC observation. Bars = 50 µm.

Figure S3 Lugol staining of 5-day-old RAM in WT and atzrf1a;b mutant via DIC observation. Bars = 20 µm.

Figure S4 Comparison of RAM development between WT and atzrf1a;b mutant at 21DAS via Lugol staining. Bars = 50 µm.

Figure S5 Cotyledon vein pattern in 10-day-old seedlings of WT and atzrf1a;b mutant via DIC observation. The samples were cleared in chloral hydrate solution. Bars = 1 mm.

Figure S6 Other defective embryogenesis in atzrf1a;b mutant. **(A-B)** Globular stage embryos in WT; **(C-K)** Embryos of different stages in atzrf1a;b mutant. Bars = 20 µm except 50 µm in **(H)** to **(J)**.

Table S1 List of primers used in this study.

Table S2 Relative expression levels of some RAM-regulating genes in atzrf1a;b compared with WT.

Table S3 Relative expression levels of auxin-responsive genes in atzrf1a;b compared with WT.
