# Supplementary figures and images for: Arabidopsis ZUOTIN RELATED FACTOR1 Proteins Are Required for Proper Embryonic and Post-Embryonic Root Development
Source: Front Plant Sci. 2019 Nov 22;10:1498. doi: 10.3389/fpls.2019.01498 (PMC6882920; doi:10.3389/fpls.2019.01498)

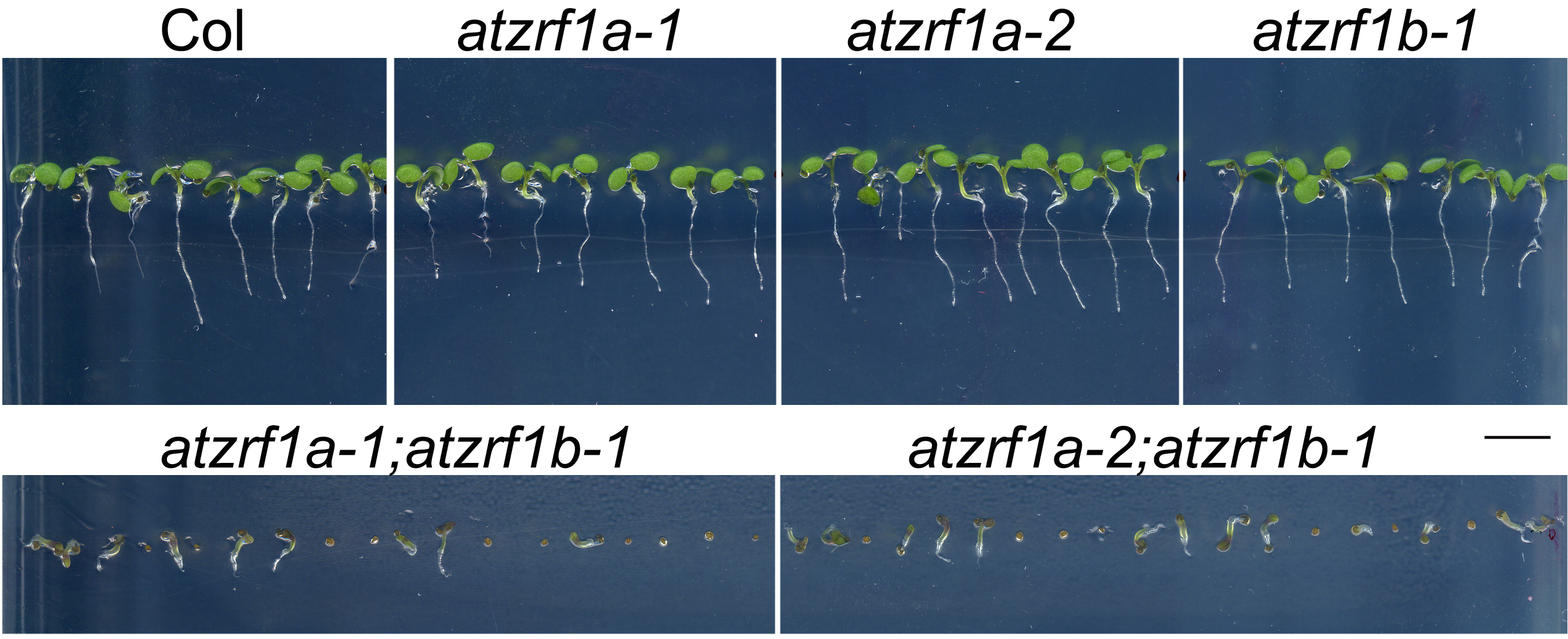

Supplement: Figure S1 — The representative root phenotype in 5-day-old atzrf1 single and double mutants. The atzrf1a-1;b-1 and atzrf1a-2;atzrf1b-1 double mutants displayed the same extremely short-root root phenotype, whereas WT and atzrf1 single mutants displayed the normal root growth. Bar = 1 cm. [file Image_1.tif]

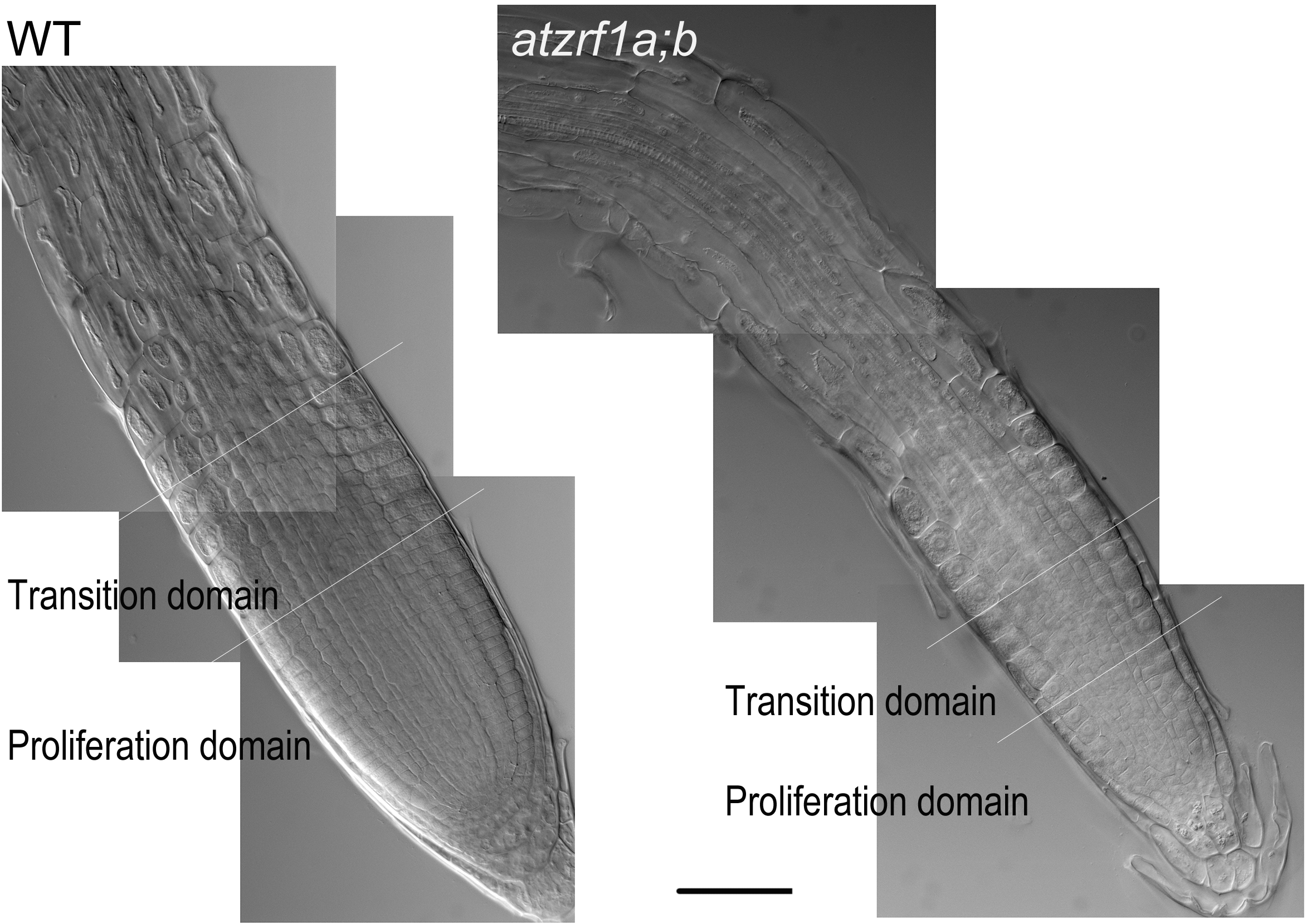

Supplement: Figure S2 — 7-day-old root phenotype in WT and atzrf1a;b mutant via DIC observation. Bars = 50 µm. [file Image_2.tif]

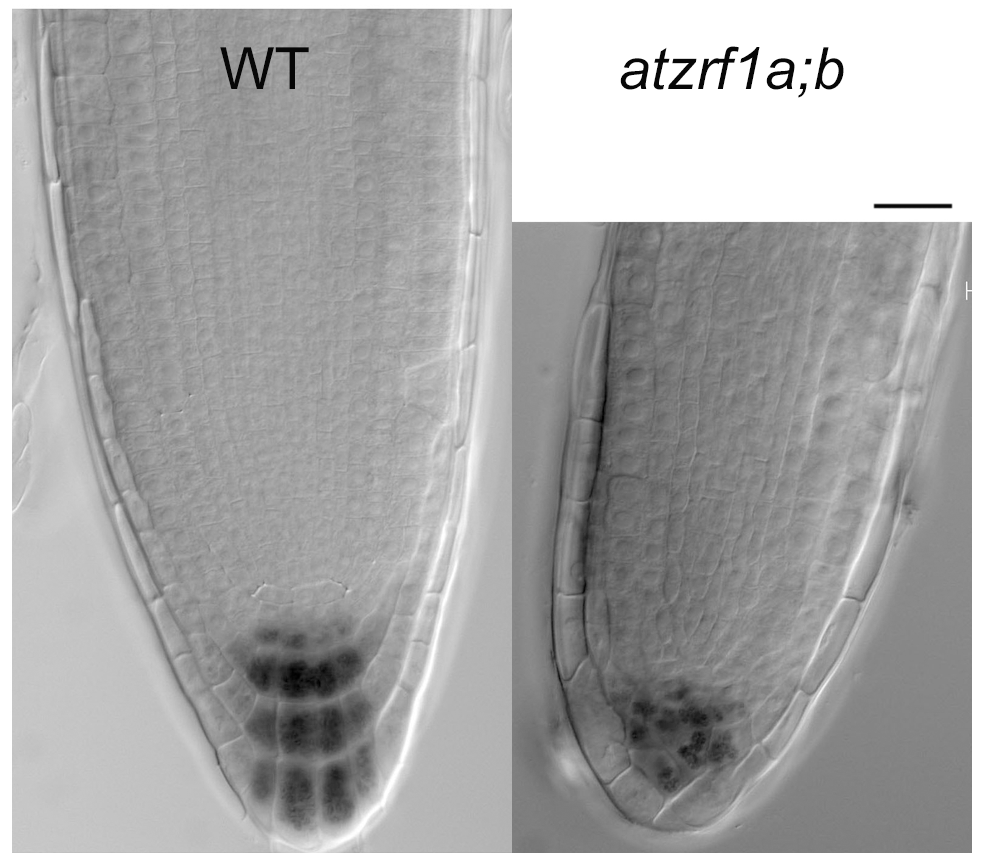

Supplement: Figure S3 — Lugol staining of 5-day-old RAM in WT and atzrf1a;b mutant via DIC observation. Bars = 20 µm. [file Image_3.tif]

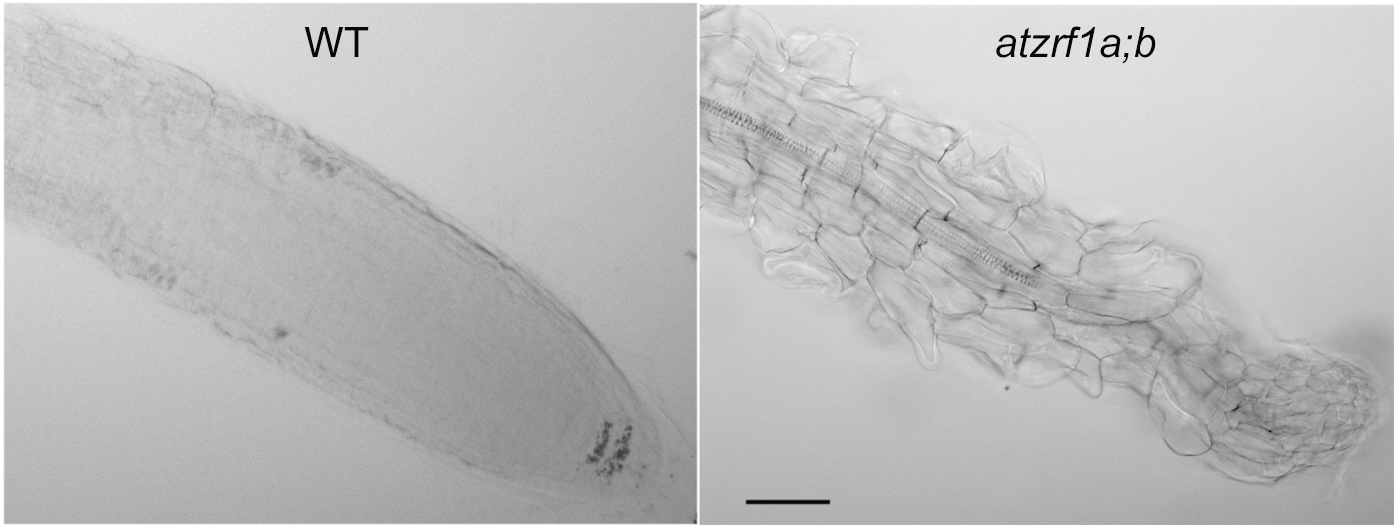

Supplement: Figure S4 — Comparison of RAM development between WT and atzrf1a;b mutant at 21DAS via Lugol staining. Bars = 50 µm. [file Image_4.tif]

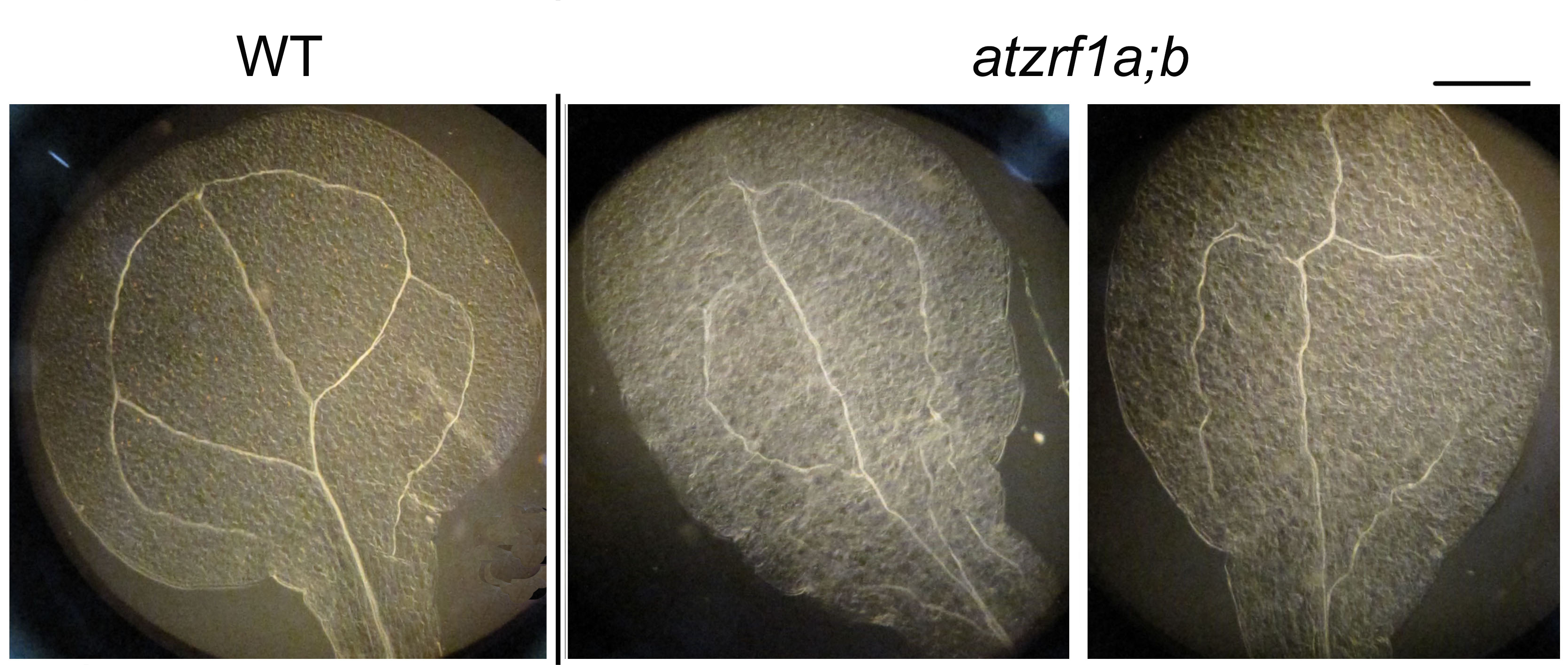

Supplement: Figure S5 — Cotyledon vein pattern in 10-day-old seedlings of WT and atzrf1a;b mutant via DIC observation. The samples were cleared in chloral hydrate solution. Bars = 1 mm. [file Image_5.tif]

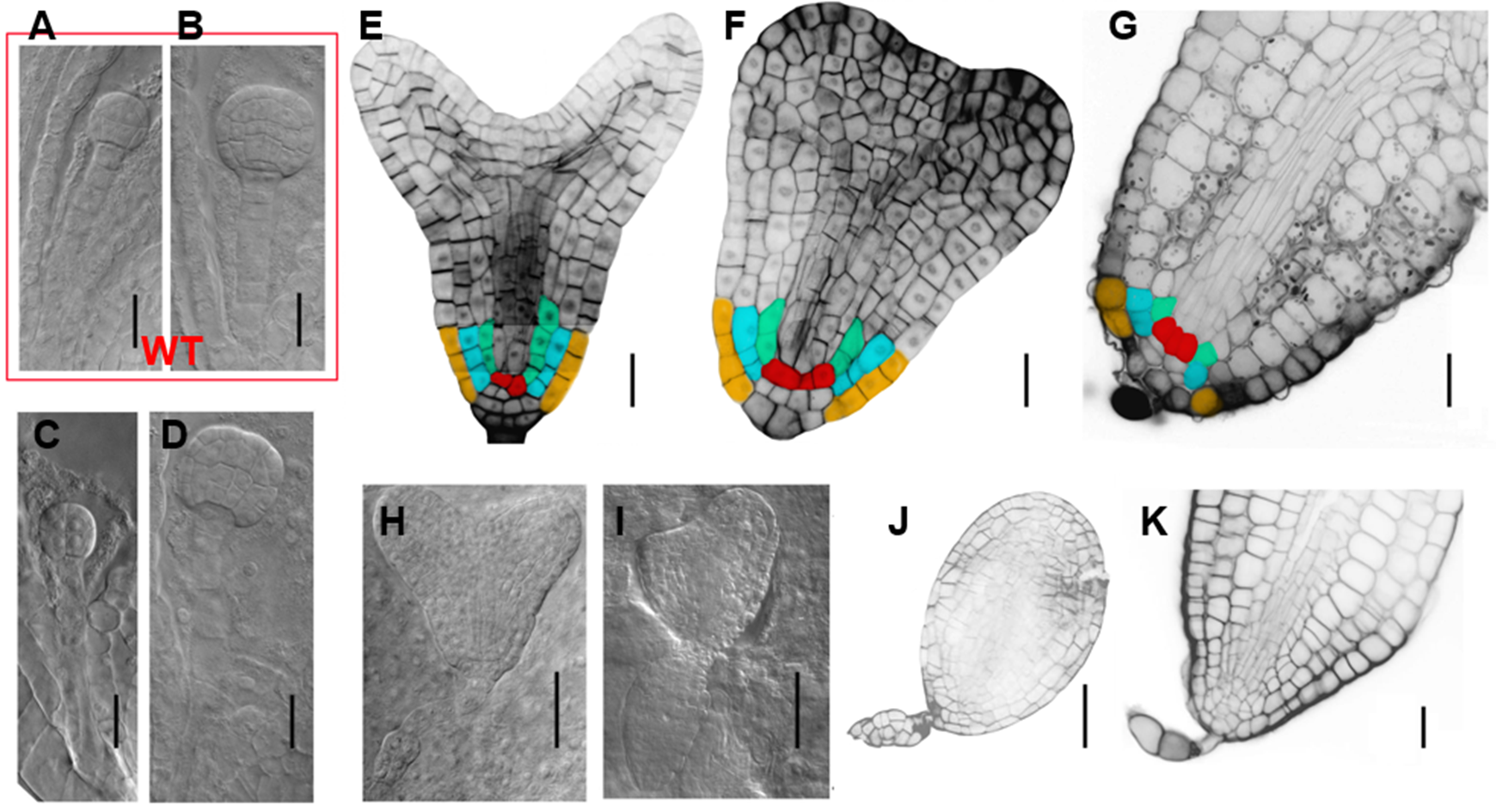

Supplement: Figure S6 — Other defective embryogenesis in atzrf1a;b mutant. (A-B) Globular stage embryos in WT; (C-K) Embryos of different stages in atzrf1a;b mutant. Bars = 20 µm except 50 µm in (H) to (J). [file Image_6.tif]
